# STRESS RESPONSE DURING COGNITIVE TASKS USING EEGS

**DOI:** 10.1093/geroni/igae098.2259

**Published:** 2024-12-31

**Authors:** Kristine Vergara, Mohammad Khalil, Kiran George

**Affiliations:** California State University Fullerton, Fullerton, California, United States; California State University Fullerton, Fullerton, California, United States; California State University Fullerton, Fullerton, California, United States

## Abstract

Abstract - Drawing upon literature indicating the potential positive effects of engaging in cognitive tasks, particularly memory games, on the mental well-being of older adults, this study aims to investigate the relationship between cognitive tasks and stress responses among the aging population. We hypothesize that older adult participants who engage in memory games will exhibit varied stress responses based on the difficulty levels of the tasks. In this preliminary investigation, we present initial findings from a subset of participants (N = 7) aged 65 and above, focusing on their EEG readings collected during memory game sessions using the g.Nautilus Research headset. Our preliminary analysis suggests a correlation between increased task difficulty and elevated theta and delta brain wave activity, indicating heightened stress responses during more challenging cognitive tasks. Ongoing data processing using MATLAB will allow for a more comprehensive comparison of EEG signals between individuals with and without significant health conditions, further elucidating the relationship between cognitive tasks and stress levels among older adults. These preliminary insights pave the way for a deeper understanding of the impact of cognitive activities on stress responses in this population, with implications for the development of targeted interventions within healthcare and mental wellness frameworks. Keywords: Aging, stress, cognitive tasks, Electroencephalography (EEG), MATLAB

